# Using Geographic Information Systems (GIS) to assess the role of the built environment in influencing obesity: a glossary

**DOI:** 10.1186/1479-5868-8-71

**Published:** 2011-07-01

**Authors:** Lukar E Thornton, Jamie R Pearce, Anne M Kavanagh

**Affiliations:** 1 Centre for Physical Activity and Nutrition Research, School of Exercise and Nutrition Sciences, Deakin University, 221 Burwood Highway, Burwood, Victoria, 3125, Australia; 2 Institute of Geography, School of Geosciences, The University of Edinburgh, Edinburgh, EH8 9XP, UK; 3 Centre for Women's Health, Gender and Society, Melbourne School of Population Health, The University of Melbourne, Parkville, Victoria, 3010, Australia

## Abstract

Features of the built environment are increasingly being recognised as potentially important determinants of obesity. This has come about, in part, because of advances in methodological tools such as Geographic Information Systems (GIS). GIS has made the procurement of data related to the built environment easier and given researchers the flexibility to create a new generation of environmental exposure measures such as the travel time to the nearest supermarket or calculations of the amount of neighbourhood greenspace. Given the rapid advances in the availability of GIS data and the relative ease of use of GIS software, a glossary on the use of GIS to assess the built environment is timely. As a case study, we draw on aspects the food and physical activity environments as they might apply to obesity, to define key GIS terms related to data collection, concepts, and the measurement of environmental features.

## Background

The role of the built environment in explaining the spatial patterning of obesity has recently received considerable attention in the public health and epidemiology literature [1,2]. The built environment comprises of urban design, land use, and transportation systems [3]. Research in this field has shown that features of the built environment exert an influence on physical and mental health as well as health behaviours, independently of the socio-demographic characteristics of the people living in these places [4-6]. For instance, researchers have evaluated whether aspects of the food environment including access to supermarkets, convenience stores, and fast food outlets are associated with body mass index (BMI) [7,8]. Similarly, other features of the built environment that influence obesity through the promotion of physical activity include street connectivity, transport infrastructure, and the location and quality of community resources (e.g. parks and schools) [9,10]. Built environments that encourage unhealthy eating or are not conducive to physical activity are often termed obesogenic [11].

Public health researchers with an interest in the built environment have benefited from the emergence of Geographic Information Systems (GIS) technology [12]. GIS offers the opportunity to integrate spatial information from a range of disparate sources into a single framework, and to use these data to develop precise measures of the built environment. The tools available within a GIS also enable precise spatial measures to be derived such as the road distance from a household location to the nearest supermarket or calculations of the amount of neighbourhood greenspace.

This glossary introduces unfamiliar users to key terminology and some of the ways in which GIS can be utilised to measure and represent features of the built environment that may relate to obesity as well as highlighting some basic methodological issues. The terms covered are restricted to those where GIS has, or has the potential to assist in developing more precise measures of the built environment. Text in italics refers terms defined elsewhere in the glossary. Terms are divided into three key categories: 1) data collection; 2) concepts; and 3) measurement.

## Data collection

### Data acquisition

One of the greatest challenges facing GIS users is the acquisition of detailed data sources that contain locational and attribute information on the built environment. Spatial data can be acquired using primary or secondary data collection methods. Primary data are often collected using two common methods: 1) "psychometric"[13-15] based on surveys of individuals who report on characteristics of the environmental feature of interest; and/or 2) "ecometric"[16,17] though direct or "systematic social" observations undertaken by fieldwork auditors who visit neighbourhoods to make observations or to complete an audit tool [18]. More recently, tools that enable the direct integration of collected spatial data into GIS have been developed including *Global Positioning Systems (GPS)*[19] and remote sensing (captured remotely using satellites to identify green space, topography etc.). Secondary spatial data are collected by external sources and include administrative data (e.g. from a census), commercial data (e.g. from market research companies), internet resources (e.g. company websites or Google street view), and phone directories (e.g. yellow pages). Commercial data are increasingly being acquired by researchers as a key data source for identifying features of the built environment [20-22]. Compared to primary data these, and other secondary data sources, may be relatively cost-effective to obtain and can usually be sourced for specific study areas or across a large geographical area (e.g. nationwide). Where secondary data are utilised, it is important to record the steps taken in this process (in the form of metadata) so future users can accurately interpret and use these data and that the process can be replicated by other researchers. A key drawback of secondary data sources is that they are often not designed for the analytical purposes for which they are being used and therefore may not entirely meet the needs of the researcher. Therefore, in order to ensure their accuracy, validation against primary data is often preferable. Discordance between data collected in the field (primary data) and secondary data are mainly due to three possible errors:[23] 1) facilities included in the commercial database are not found in the field; 2) facilities are included in the commercial database but not considered to be the same service type when identified in the field; 3) facilities found in the field were not in the commercial database. Specific results on the accuracy of secondary data sources have previously been reported for physical activity facilities [23,24] and the food environment [24-27]. To summarise, findings suggest most sources of secondary data have sufficient error to potentially introduce bias into analyses. Both primary and secondary data often require manual *geocoding *to transpose the data into a GIS compatible format.

### Geocoding

Geocoding is the process of matching raw address information (e.g. the household addresses of study participants or the addresses of neighbourhood resources such as supermarkets) with a digital spatial dataset that includes all addresses within the area of interest mapped to latitude and longitude coordinates [28]. Geocoding is often preceded by *data acquisition *whereby data are acquired from primary or secondary sources. Geocoding is prone to a number of errors which can bias estimates of the associations between the built environment and health [23,29,30]. The first source of error relates to the match rate which is the percentage of addresses that are successfully geocoded. Higher match rates are achieved when the raw address file is accurate and the digital data set is comprehensive and regularly updated. Low match rates may occur because of incomplete address information and errors such as incorrect street suffixes, mis-spelling of street names, suburbs, and postal area information. Second, even when high match rates are achieved, addresses may be geocoded to the incorrect location. This error may arise because of inaccuracies in the raw address and spatial digital files or the program settings (i.e. the criteria used to define a match such as sensitivity to spelling of street names).

### Global Positioning System (GPS)

A Global Position System (GPS) is a device that uses a satellite system to pinpoint a stationary location on the earth to a latitude and longitude coordinate. In environment and health work, it is a valuable tool for field auditors that can facilitate the accurate and precise primary *data acquisition *of the location of features within the built environment such as food stores, parks or outdoor advertising [31]. GPS devices also enable investigators to track the mobility patterns of individuals through the environment to develop measures of their travel routes and *activity spaces *[32]. These technologies have recently been coupled with devices such as accelerometers (that provide objective measures of physical activity) so that the precise location where the physical activity is occurring is also captured [33,34]. Given the high cost of the equipment, these data are often costly to collect, especially when seeking sufficient numbers to power epidemiological analyses. Further, GPS technologies are at the developmental stage and challenges remain including signal loss, slow location detection, precision of the device, battery power, and study participants forgetting to switch on the device. These factors may affect the completeness and accuracy of the GPS data. However, to aid new users, data collection and cleaning protocols to reduce the severity of these potential issues have been developed [19,34,35].

## Concepts

### Accessibility

Accessibility refers to the ease of access to a particular neighbourhood feature with more accessible destinations having lower travel costs in terms of distance, time, and/or financial resources [36]. Accessibility to built environment features is not only determined by their distribution across space but also by mobility factors such as private vehicle ownership or public transportation networks [36-38]. Handy and Niemeier [36] suggest three categories of accessibility measures: 1) cumulative opportunity measures which is simply a count of features within a given distance with an equal weight applied to all occurrences of a specific feature; 2) gravity based models where features are weighted by factors such as the size of the destination or travel cost; and 3) random utility-based measures where theory is used to inform the probability of an individual making a particular choice depending on the attributes assigned to that choice (e.g. attractive of destination or potential travel barriers) relative to all choices. An alternate accessibility measure that incorporates a temporal dimension has been proposed by Kwan [39]. Space-time measures' incorporate the constraints imposed by the fixed locations an individual must visit during the day (e.g. location of work-place, child's school) when determining potential accessibility to discretionary locations that an individual may visit (e.g. supermarket) (see also *activity space*). Greater locational access to neighbourhood features may improve or worsen the health-related behaviours of local residents. For example, high levels of accessibility to a greengrocer or large supermarket may better enable the purchase of fresh fruits and vegetables while greater accessibility to outlets selling fast food may encourage the consumption of fast food at levels that are damaging to health. Traditionally, accessibility has been rather simply measured through the presence or absence of a resource in a particular locality because these data were readily available. These measures assume equal exposure for each person within the area unit irrespective of where they live in that unit, the amount of time that they spend in the area, and their ability to travel within and beyond the boundary of the administrative unit. GIS has improved measurements of accessibility by enabling the creation of more refined individual-level metrics such as *density *within *buffers *from a household location, *proximity *based on *network distance, activity-spaces*, and continuous surfaces of accessibility such as *Kernel density estimations*.

### Scale

Selecting an appropriate spatial scale for measuring features of the built environment using GIS is an important prerequisite for research into neighbourhood influences on health [40]. Different characteristics of the built environment are likely to influence the health and/or behaviours of local residents at various spatial scales. For instance, it could be argued that the influence of access to a local corner store may have a greater effect on the local resident population (i.e. neighbourhood), whereas the availability of a large supermarket is likely to extend to a wider geographical extent. Further, the most appropriate spatial scale for capturing the neighbourhood feature could be influenced by the socio-demographic characteristics of the study population (e.g. children compared with elderly) as an individual's capacity and motivation to travel longer distances is likely to be affected by personal mobility and *activity space*. Other considerations include whether the area of interest is urban or rural as environmental exposures in an urban setting may be confined to a more localised population, whereas rural features are likely to be utilised by those from a larger spatial region [41]. Thus, for studies examining exposures related to the built environment no one scale can be recommended. Ideally the selection of the spatial scale will be informed by the theoretical understanding of the processes that link the neighbourhood characteristic(s) to health [41-45]. However, it has been recognised that relevant theory does not always exist [44]. In situations where this is so, for example amongst studies examining walkability, the result is that buffer sizes used for measures of walkability have varied from between 100 metres to 1 mile (approximately 1600 metres) or to even larger areas defined by the boundaries of administrative units [46]. Decisions regarding scale are external to GIS, however the advantage of GIS is that many plausible scales might be investigated.

## Measurement

### Activity space

**Figure 1:**
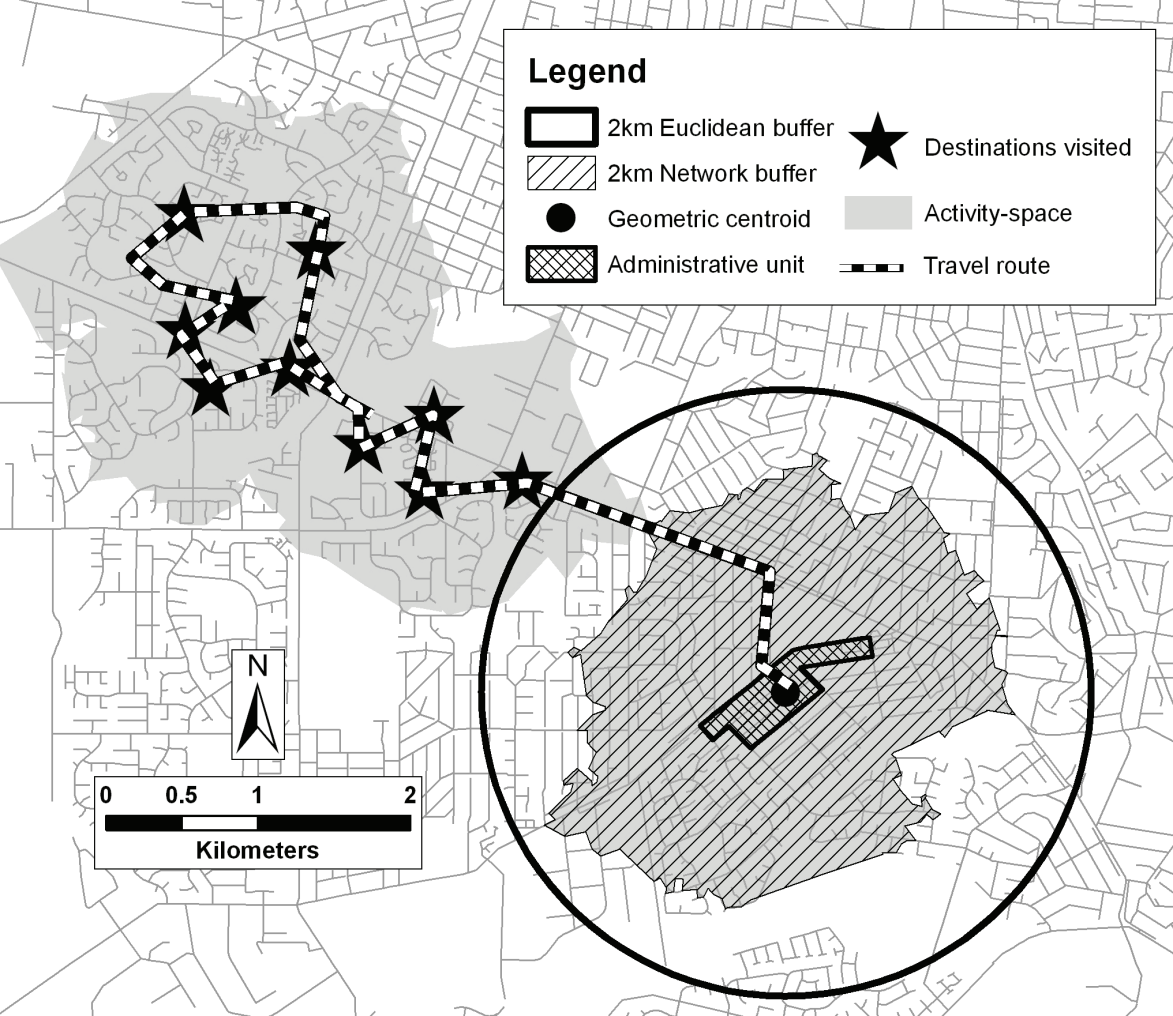
**Examples of measures of accessibility**. Terms: accessibility, activity space, buffer, centroid (geometric within an administrative unit), network distance. This figure demonstrates the different approaches to measuring boundaries of spatial units used for *accessibility *measures such as *density*. Firstly, the point from which the measures will be taken is defined; in this case a geometric *centroid *of an administrative unit (census collector district, the smallest administrative spatial unit in Australia) is calculated. From this point, two *buffers *are drawn; the first using Euclidean (straight-line) distance and the other using *network distance*. The third spatial unit relates to *activity space*. This relates to an individual's travel patterns over a course of a day with the destinations visited and the travel routes mapped (both of which can be captured using a *Global Position System (GPS) *device). A *buffer *is also placed around these to capture exposures nearby to the visited locations and also nearby to their household (represented by the geometric centroid).

An activity space represents all locations visited by an individual within a specified time period. Activity spaces are important to consider because residents often engage in a multitude of activities outside of their local environment. The geographical extent of an activity space is likely to be determined by both environmental and individual-level factors [47-49]. For instance, the *proximity *of resources dictates how far an individual is required to travel to reach these while at the individual-level factors such as age, gender, access to a motor vehicle, and/or perception of distance and safety all influence the ability and willingness of an individual to access the resource. Mapping an individual's activity space potentially provides a more precise reflection of their true contextual exposures and therefore improves specificity between the exposure and behavioural or health outcomes. Activity spaces may be captured through personal diaries where individuals record daily activities or the use of *Global Positioning System (GPS) *devices. An individual's travel patterns can be represented as an activity space within a GIS using a variety of methods [50-52] with two examples being mapping a *buffer *around the travel routes and locations visited during the day (see Figure 1) or through 3-D visualisation which can be used to display space-time parameters that effectively represent the regularity of travel patterns [50].

### Buffer

Buffers are boundaries placed around areas (e.g. the boundary of an administrative unit) or points (e.g. a household or the *centroid *of an administrative unit) using a predefined *scale *using either a straight-line (Euclidean) or *network distance *(Figure 1). Buffers are useful for capturing all features of the built environment that surround a particular location. For example, the number of supermarkets within a buffer might be used to estimate a household's *accessibility *to supermarkets. However, limitations include the binary representation of a features (e.g. it is either considered in our out of the buffer) which can be overcome with the consideration of a fuzzy (using a decreasing weight function for distances further away) rather than sharp boundary [53]. Buffers are readily created within a GIS once the user has defined the *scale*, type (Euclidean and *network distance*), and point they are measuring from (e.g. around a household or *centroid*). These decisions should be informed by the hypothesised relationship between the exposure and outcome [45].

### Centroid (geometric and population-weighted)

A centroid is a single point, representing the 'centre', of a spatial unit (Figure 1). Centroids may be used as the point from which exposure measures are undertaken such as *proximity *estimates or the *density *of features in a *buffer*. GIS enables the identification of geometric centroids (the geographical centre) or population-weighted centroids (the point that minimises the total distance to all the residents (or households) in an area). Population-weighted centroids are particularly useful when the population is homogeneously distributed in space (such as in rural areas or larger spatial units) and where a geometric centroid will not result in a precise representation of *accessibility *for most residents. However, neither centroid measure will provide data as precise as individual-level measures (e.g. using individual household location to derive accessibility measures).

### Connectivity

**Figure 2:**
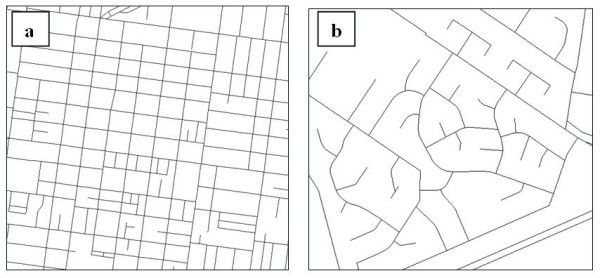
**Comparison of environments with: a) a grid street pattern with high-connectivity; b) a poorly connected street network**. Terms: accessibility, connectivity, walkability. Figure 2 demonstrates the differences between high street-network *connectivity *(Figure 2a) that would provide a more direct route between a origin and destination compared to low street-network *connectivity *with many cul-de-sacs and dead-ends (Figure 2b) which reduces the directness travel routes.

Connectivity relates to the availability and directness of travel routes used to move through a network from an origin to a destination [3,10,54]. Common approaches to the measurement and assessment of connectivity include:[54,55] 1) identifying the spacing between streets (with a tight grid formation resulting in higher connectivity); 2) assessing the amount of intersections with connecting streets that provide four or more routes choices (as opposed to t-intersections and dead-ends); and 3) comparing the *network distance *to the Euclidean distance (a network distance that is only marginally above Euclidean distance indicates a very direct route along the network). High connectivity improves *accessibility *by providing a more direct route and shortening the required travel distance (Figure 2a). Neighbourhoods with low connectivity might contain numerous cul-de-sacs, large block sizes and fewer intersections (Figure 2b). When investigating connectivity for walking purposes, it is important to also include paths used solely for pedestrian purposes as street-network databases tend to be restricted to parts of the network accessible to motor vehicles [56]. Higher levels of connectivity has been associated with greater levels of physical activity as shorter and more direct routes encourage walking for transport and reduce car dependency [10,57]. However, high street-network connectivity may also negatively impact on *walkability *by potentially increasing motor vehicle traffic on residential streets, thus reducing pedestrian safety [54]. Further, whilst connectivity may inform us about the directness of the route, it is only a single aspect related to *walkability *and, measured alone, it is unlikely to provide sufficient information to determine whether an area is considered walkable.

### Density

Density is a measure of the intensity of exposure to features of the built environment and may be an important determinant of health behaviours as it relates to the *accessibility *of potentially health promoting and health damaging environmental characteristics. Density may be expressed simply as a count of features within a specified area (e.g. total number within a postcode or a *buffer) *but is more accurately represented as the relative number of features per population (e.g. number per 10,000 people) or per geographic area (e.g. number per square kilometre). Adjustment for population or geographic area is most useful when trying to explain the distribution of features across areas as these may provide an explanation as to why some features appears in greater numbers in some areas and not others [38]. For parks and open spaces, density may be reflected by the count of features or the geographic area of these features. Continuous measures of density assume that the association between the feature and the health outcome of interest increases linearly with each unit increase, however it is possible that once the density of a feature reaches a certain threshold further increases in density may no longer be linearly associated with the outcome. For example, having access to multiple McDonalds restaurants means *accessibility *is increased through greater exposure but this exposure is to the same product so it does not improve your product choice or variety.

### Kernel density estimation

**Figure 3:**
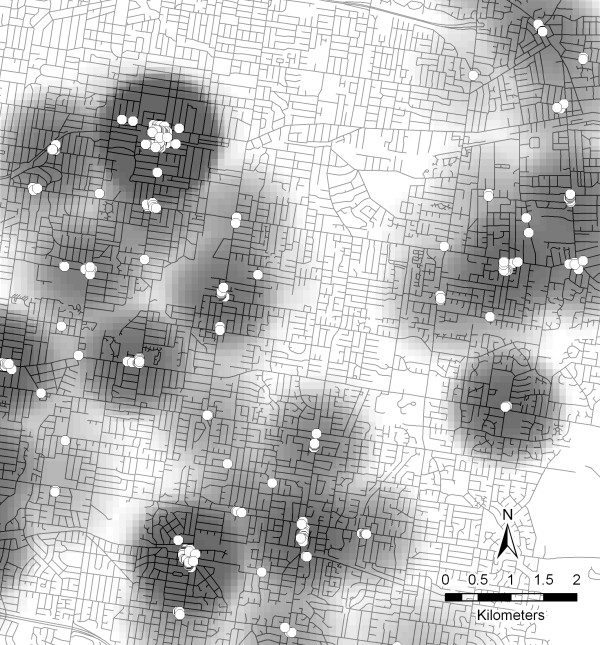
**An example of a map resulting from kernel density estimation**. Terms: accessibility, kernel density estimation. Figure 3 demonstrates the output map resulting from kernel density estimation with the kernel size set at two kilometres. Darker areas indicate where resources are more densely located while lighter colouring relate to areas with reduced accessibility.

Kernel density estimation is a technique for transforming point data to a continuous density surface map whereby the density of a feature can be estimated for any point on the surface (Figure 3). To create Kernel density estimates, the entire study region is partitioned into grid cells of a predetermined size. The kernels (which are usually circular in shape with the radius defined by the user) are then placed around the centroid of each cell (or alternatively the crossing point of the grid cells). For each feature within the kernel, weights are assigned as a defined function of distance from the geometric *centroid *of the kernel. This results in a density value being assigned to each cell so that density values can be calculated across the whole study region. In studies of the built environment and health, the technique has previously been used to calculate robust measures of exposure to one or more environmental features (e.g. access to food outlets or recreational facilities) across a study area [58-62]. This approach is advantageous compared to traditional *density *measures because a resource that is located closer to the grid cell is assigned more weight than resources that are located further away, with the weight approaching zero at the boundary of the kernel. Thus, the transition to the boundary represents a fuzzy rather than sharp boundary [53] and can be utilised as a gravity-based measure of *accessibility *[36].

### Land use and land use mix

Broad categories of typical land uses include (but are not limited to) residential, office, commercial, industrial, and recreational and there are multiple existing measures of land use mix [10,46,55,63-65]. A specific example of a measure of land use mix is the "dissimilarity index" which measures the evenness of the distribution of a range of land uses across a predefined geographical area [55,65]. Areas with low land use mix are homogenous in terms of the uses of space (e.g. area is mostly residential or commercial) whereas areas with a high land use mix have a greater variety of land uses (such as recreational, industrial, commercial, educational etc.). Residents of neighbourhoods with a mixture of land uses have higher *accessibility *to features they may wish to visit and consequently a more confined *activity space*. Some research has demonstrated people living in neighbourhoods with a high land use mix are more likely to be physically active (through active travel) and have a lower likelihood of overweight or obesity [64] however others have shown that the presence of specific walkable land uses (e.g. parks) may be more important than having equal amounts of different land uses in an area [63]. GIS enables the integration of land use data from a range of sources from which the user can develop measures of land use mix.

### Network distance

Network analysis enables the measurement of the distance between an origin and destination along a network of lines which can include road, public transportation, pedestrian and/or cycling network paths. Because distance is measured along the transportation network rather than as Euclidean (straight-line) distance, network distance can provide a more precise measure of *accessibility *(Figure 1). Within built environments, the network travel distance required to reach a destination may be significantly greater than the straight line distance due to features related to the built environment (e.g. the presence of buildings), natural barriers (e.g. rivers or steep hills), and characteristics of the network itself (e.g. cul-de-sacs, one-way streets). Network distance measures can be readily calculated within GIS provided that accurate network data are available. Measures of travel time can also be derived (e.g. the number of minutes required to travel from a participant's house to the closest swimming pool) in a GIS using information on network distance and the average speed of travel along each segment of the network. It is also feasible to develop more sophisticated measures of travel time that incorporate factors such as traffic density, traffic signals, road surface, and topography, each of which would improve estimates of *accessibility*.

### Proximity

Proximity, or closest facility analysis, is an important indicator of *accessibility *and is used to determine which feature (e.g. gymnasium) is closest to a particular point (e.g. household location) and/or the actual distance to the nearest feature. Proximity is important because *accessibility *is increased when features are closer thus potentially influencing their contribution to health behaviours. Proximity can be measured using Euclidean distance, *network distance*, or the estimated travel time along a network. Proximity measures derived from network analysis are based on a least-cost analysis; that is, the shortest distance or time from an origin to a destination. As the actual travel routes for study subjects are not usually known, least-cost analysis is considered the best approximation as it assumes the subject would use the shortest travel route (or quickest if travel time estimations are used).

### Walkability

Walkability can be conceptualised in terms of four key components: functionality, safety, aesthetics, and destinations [66]. Each component of walkability has a number of sub-categories that can be created within GIS. For example, c*onnectivity *is one feature of functionality while *land use mix *relates to the presence and variety of destinations. Whilst there are a number of potential variants to measures of walkability,[46,66,67] to date these have not been consistently measured. Residents living in environments considered more 'walkable' have been linked to increased levels of physical activity [57] and lower BMI [64]. Specifically, levels of walking may be enhanced through higher pedestrian-network *connectivity *and greater *land use mix*. Since walking is the most common form of physical activity, identifying the key attributes of the physical environment that contribute to walking is of considerable public health importance. Whilst *data acquisition *for some walkability measures such as presence of traffic control devices or walking paths can often be sourced from existing GIS databases, others related to aesthetics such as litter and graffiti tend to require observers to specifically audit areas [66,68,69].

## Discussion

Geographic Information tools have been described as one of six innovations at the frontier of social science research and has important application to studies of the built environment and health [70]. Coupled with new advances in epidemiology, such as multilevel statistics and spatial analysis methods, GIS has the potential to contribute to the advancement of our understanding of the importance of the built environment for obesity. However, important methodological challenges remain relating to data collection, GIS concepts, and the measurement of the built environment [32,71-73]. This glossary provides public health researchers with an introduction to GIS; its potential to contribute to our understanding of the built environment and obesity; and the basic concepts and methods related to using GIS. Further, the correct and consistent use is aided by protocols such as those already developed by Forsyth [74] and the ever growing collections of up-to-date web-based resources including the International Physical Activity and the Environment Network http://www.ipenproject.org/, The Global Positioning Systems in Health Research Network http://www.gps-hrn.org/ and the US National Cancer Institute: Measures of the Food Environment https://riskfactor.cancer.gov/mfe. Nonetheless, a familiarisation of key terms is not a substitute for an understanding of geographical and mapping principles (e.g. map projections and edge effects) and the need for theoretically-informed rather than data-driven analytical approaches [75].

## Competing interests

The authors declare that they have no competing interests.

## Authors' contributions

LET drove the design of this study and wrote the first draft of this paper. JRP and AMK contributed to the study design and the redrafting of the paper. All authors read and approved the final version of the final paper.
